# A new species of *Oileus* Kaup (Coleoptera, Passalidae) from Guatemala, with a key to the species of the genus

**DOI:** 10.3897/zookeys.194.2963

**Published:** 2012-05-17

**Authors:** Enio B. Cano, Jack C. Schuster

**Affiliations:** 1 Universidad del Valle de Guatemala, Apartado Postal 82, 01901, Guatemala, Guatemala; 2 Museo de Historia Natural, Escuela de Biología, Universidad de San Carlos de Guatemala, Calle Mariscal Cruz, 1-56 zona 10, Guatemala, Guatemala

**Keywords:** Passalidae, *Oileus*, Guatemala, Quiché, cloud forest

## Abstract

*Oileus gasparilomi* **sp. n.** is described from nine specimens from the mountains of Quiché in Guatemala, from cloud forest at 1795 m elevation. This represents the second species of the genus for Guatemala, differing from the closely related Mexican species *Oileus rimator* (Truqui) in having a straight anterior clypeal border, the postocular sulcus divided by a keel and the pronotum almost straight (not clearly bilobed). A key to the species of *Oileus* is given.

## Abstract (de)

Se describe *Oileus gasparilomi* **sp. n.** de un bosque nuboso a 1795 m, de las montañas de Quiché en Guatemala. Esta es la segunda especie del género conocida para Guatemala y se separa de la especie mexicana más relacionada, *Oileus rimator* (Truqui), porque posee el borde anterior del clípeo recto, el surco postocular dividido por una quilla y el pronoto casi recto (no claramente bilobado). Se provee una clave para las especies *Oileus*.

## Introduction

The genus *Oileus* Kaup consists of five species (Quintero and Reyes-Castillo 1983); four endemic to México (*Oileus rimator* (Truqui), *Oileus heros* (Truqui), *Oileus nonstriatus* (Dibb) and *Oileus bifidus* (Zang)), and one (*Oileus sargi* (Kaup)) widely distributed from Chiapas to Panamá (Quintero and Reyes-Castillo 1983) and Colombia (Schuster 1993). Recent collections in previously inaccessible localities (due to civil war) in Quiché department of Guatemala provided a second species in the genus for that country which proved an undescribed species. Here we describe and illustrate the new species and provide a key to the six species of the genus *Oileus*.

## Methods

For terminology we follow Reyes-Castillo (1970), except for male genitalia where we follow Boucher (2005). Measurements were taken with a vernier caliper except antennae and eyes which were measured with an ocular micrometer in a Wild Heerbrugg M3B stereomicroscope. The total length was measured from the tip of mandibles to the tip of elytra. Drawings were made with the help of a drawing tube in a Wild Heerbrugg M3B stereomicroscope. Digital photographs were taken with a Canon Rebel T1i, and edited in Digital Image Pro 9. Locations of the specimens of the new species are as follows: Arthropod Collection of Universidad del Valle de Guatemala, Guatemala (UVGC); Museo de Historia Natural, Escuela de Biología, Universidad de San Carlos de Guatemala (USAC); Instituto de Ecología, Xalapa, Veracruz, México (IEXA); and Instituto de Biología, Universidad Nacional Autónoma de México (IBUNAM).

## Systematics

### *Oileus* Kaup, 1869

The genus was revised by Quintero and Reyes-Castillo (1983) and is characterized by the wide antennal lamellae (equal or subequal to each other), presence of the frontoclypeal suture, frons without internal tubercles, frontal fossae glabrous, and the presence of a small basal tubercle on internal surface of both mandibles; although Boucher (2005) observed just one autapomorphy present in the aedeagus: ventrodorsal basal sclerotizations of the phallus. Interestingly, the genus includes both species with well developed and reduced wings.

#### Oileus gasparilomi

Cano & Schuster sp. n.

urn:lsid:zoobank.org:act:7998265B-1510-4F1E-9CFB-58974C869BB7

http://species-id.net/wiki/Oileus_gasparilomi

Figures 1 7

##### Material examined.

Nine specimens.

##### Type material. 

Holotype male, pinned: GUATEMALA: Quiché Dept., Municipio de Chajul, aldea Chel, camino a Cabá, 15°38'57.10"N, 91°01'10.25"W (DMS), 1795m, bosque nuboso, VI 2008. Col. A. González-Madrid (UVGC).

Paratypes. five males and three females, same data as holotype, pinned (UVGC, USAC, IEXA and IBUNAM).

##### Diagnosis.

*Oileus gasparilomi* is a macropterous species, closely related to the *Oileus rimator* complex of species. The clypeus with anterior border straight, the postocular sulcus divided by a keel and the pronotum not clearly bilobed (almost straight) separates the new species from *Oileus rimator*. Ventrodorsal basal sclerotizations of the phallus (autapomorphy of the genus *Oileus* according to Boucher (2005)) is present in dorsal view of the aedeagus (Figure 4).

**Figures 1–4.**
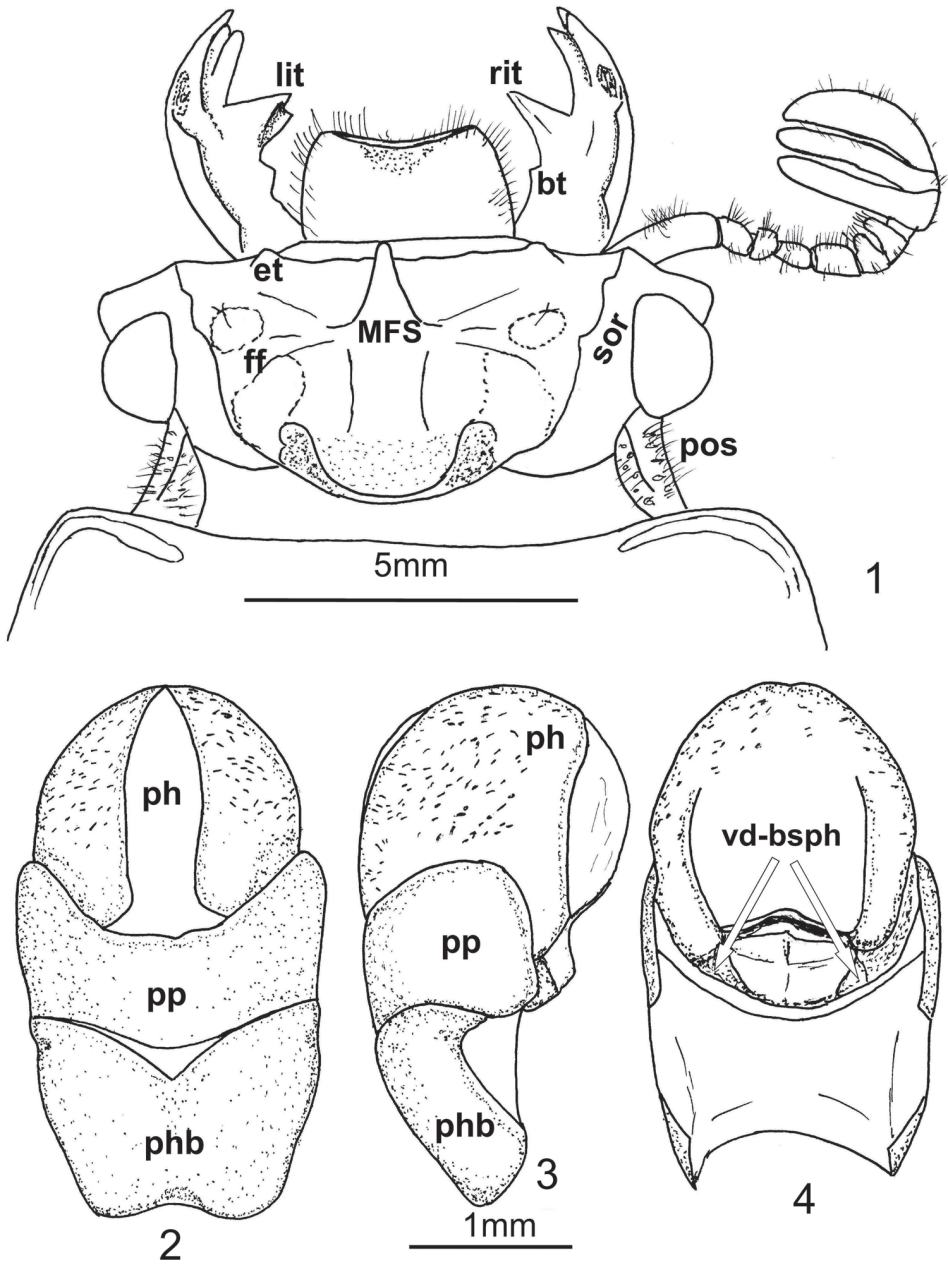
*Oileus gasparilomi* sp. n., holotype **1** head **lit** left internal teeth of mandible **rit** right internal teeth of mandible **bt** basal tubercle of mandible **et** external tubercle **MFS** Median Frontal Structure **sor** supraorbital ridge **ff** frontal fossae **pos** postocular sulcus **2–4** aedeagus **2** ventral view **3** lateral view **4**  dorsal view **ph** phallus **pp** parameres **phb** phallobase **vd-bsph** ventrodorsal basal sclerotizations of phallus

**Figures 5–7.**
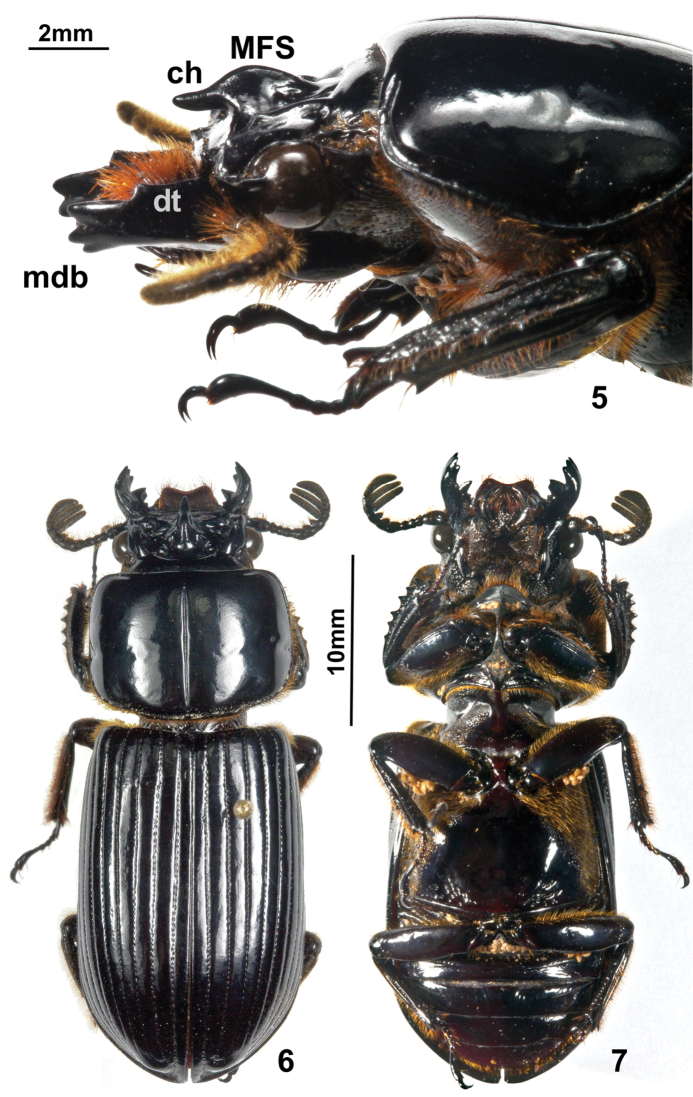
*Oileus gasparilomi* sp. n., paratype (USAC) **5** lateral view of head and pronotum **MFS** Median Frontal Structure **ch** center horn **mdb** mandible **dt** dorsal tooth of mandible **6** habitus in dorsal view **7** habitus in ventral view

**Description.** Head. Anterior border of labrum concave, dorsal surface granular. Clypeus inclined, anterior border straight, posterior border crenulate. Frontoclypeal suture slightly concave at center, external tubercles rounded. Frontal area inclined, smooth and shiny in front of Median Frontal Structure (MFS); frontal ridges feebly developed, inner tubercles absent. Frontal fossae glabrous and smooth, composed of three separated small fossae: the anterior separated from the slightly bigger medial one by a keel than runs laterally from the MFS, and this separated from the posterior by an incomplete keel. MFS of “striatopunctatus” type (see Reyes-Castillo 1970); sides of base of MFS subparallel, as wide as rest of MFS; center horn long with apex largely free and slightly directed upward, surpassing the posterior margin of clypeus (Figure 5); without dorsal groove. Supraorbital ridge bituberculate, anterior tubercle larger than posterior; posterior half of ridge elongate, with a small posterolateral impression. Canthus with apex rounded, apex forming a right angle. Eyes large, width = 0.98 mm (each eye). Head width (including eyes) = 9.2 mm. Ratio of sums of both eyes widths/total head width = 0.21; postocular sulcus wide, punctate and setose, divided by a central longitudinal keel. Ligula markedly protuberant medially, with central tooth small; setose punctations present laterally and posteriorly. Lateral lobes of mentum opaque towards the borders, with abundant setiferous punctations. Base of mentum medially glabrous and shiny; anterior central border convex; basal fossae oval, punctate-setose and opaque. Hypostomal process long, wide. Infraocular ridge present; proximal area concave and striate, basal area rounded, external area punctate-pubescent. Mandibles with three apical teeth (Figure 5); internal teeth wide, short, bifid in left mandible; both mandibles with a small basal tubercle (Figure 1); dorsal tooth occupies half length of the mandible; internal face smooth. Antennal club (Figures 1, 6 and 7) with the three segments very wide and subequal (width of penultimate segment = 3.5 mm).

Thorax. Anterior border of pronotum not clearly emarginate medially, almost straight. Lateral fossae of pronotum without coarse punctations. Pronotum with marginal groove narrow and smooth; anterior angles rounded. Pronotum with sparse, small and shallow punctations, moderately visible at low magnification. Mesosternum with lateral depression elongate, with elongate punctations, setose, with external border opaque. Mesepisternum with large opaque area. Metasternum laterally with dense setiferous punctations; disk shiny, delimited by 16–19 (up to 34) rugose punctations; marginal fossa setose, with coarse elongate punctations posterior part 1.5–2 times wider than the median part.

Elytra. Striae marked with defined punctations; striae 1–4 less well marked than 9–10, interstriae 1–5 with scarce micropunctations, more abundant and visible at lower magnification on interstriae 6–10. Anterior border of elytra vertical (Figure 4); humerus with tuft of setae on interstriae 5–10.

Wings. Well developed.

Legs. Profemur with anterior-ventral groove well marked, reaching the punctate setose apical area. Mesotibia with one (or two, if a spiniform keel is present) spine(s).

Abdomen. Marginal groove incomplete, occupies 3/5 of last sternite.

Aedeagus (Figures 2–4). In ventral view, phallus spherical; parameres and phallobase not fused; parameres without a longitudinal division. In dorsal view (Figure 4), ventrodorsal basal sclerotizations of the phallus present.

Dimensions (mm) (n = 9): total length 35.75-41.80, (µ = 38.26); elytral length 20.30–22.40, (µ = 21.35); pronotal length 8.4–9.6, (µ = 9.14); pronotal width 10.7–12.0, (µ = 11.74); humeral width 11.65-12.65, (µ = 12.07).

Larva. Unknown.

##### Etymology.

Named after Gaspar Ilóm, a native hero of the novel “Men of Maize” by Miguel Ángel Asturias. The collection locality is called “mountains of Ilóm”.

##### Distribution and ecology.

The species is known only from mid-altitude cloud forest from the type locality, near Chel Village, road to Cabá in Chajul, Quiché, Guatemala. The southernmost locality of the closest relative, *Oileus rimator*, is in the northern mountains of Chiapas, Finca La Trinidad, at Municipio El Bosque (Quintero and Reyes-Castillo 1983), separated from the locality of the new species by approximately 260 km.

##### Commentaries.

Quintero and Reyes-Castillo (1983) cite one specimen of *Oileus rimator* (Truqui) from the Museum of Natural History, Paris (MNHN), identified by H.W. Bates as *“Oileus” sagittarius* Smith (an invalid, unavailable name), collected in Guatemala. They considered the locality as erroneous. The Guatemalan *Oileus gasparilomi* is closely related to the Mexican *Oileus rimator* and the specimen of the MNHN may have been confused by Quintero and Reyes-Castillo, but most probably the specimen was mislabeled. Consequently, we also consider the locality as erroneous.

### Key to the adults of known species of *Oileus*

**Table:** 

1	Lateral margin of mesosternum with abundant setae	2
–	Lateral margin of mesosternum glabrous	3
2	Anterior border of clypeus widely notched at center	*Oileus rimator* (Truqui)
–	Anterior border of clypeus straight	*Oileus gasparilomi* Cano & Schuster, sp. n.
3	Eyes reduced, kidney-shaped, at least half covered by ocular canthus; elytra oval; body length 34–48mm; Mexico	4
–	Eyes normal, globose, less than half covered by ocular canthus; elytra rectangular; body length 28–33mm; Chiapas (Mexico) to Colombia	*Oileus sargi* (Kaup)
4	Elytral striae indistinct or with feebly visible punctations; cephalic horn with dorsal longitudinal groove, extending more than one half the length of the horn; ocular canthus acute-angled	5
–	Elytral striae marked with more or less developed but always distinct punctuations; cephalic horn with dorsal longitudinal groove extending one third the length of the horn or less, or absent; ocular canthus with rounded apex, greater than 90 degrees; Oaxaca	*Oileus bifidus* (Zang)
5	Frontoclypeal suture slightly concave at center; dorsal surface of mandibles smooth; all elytral striae barely marked; body length 44–47mm; Sierra Madre Oriental	*Oileus heros* (Truqui)
–	Frontoclypeal suture straight; dorsal surface of mandibles with small punctations; elytral striae 1–2 poorly marked, 3–6 indistinct at least in anterior and posterior third; body length 42–44mm; Sierra Madre Oriental	*Oileus nonstriatus* (Dibb)

## Supplementary Material

XML Treatment for Oileus gasparilomi
